# Gold Inlays

**Published:** 1906-10-15

**Authors:** J. W. Lyons


### GOLD INLAYS.

BY J. W. LYONS, D.D.S.

Read before the Michigan Dental Association, July 10, 1906.

It was with much misgiving that I decided to accept an invitation from the committee to present a paper on the subject of the gold inlay, to this meeting of thoughful, critical and scientific men.

I wish to state at the outset, and have it thoroughly understood, that I do not present this subject with the idea that it is the method or the only method by which the dental organs may be preserved to usefulness for the patient. Whatever claim I may advance for the practice of using the gold inlay, I desire not to leave the impression that I would, in any way, discredit the use of the many other valuable filling materials and methods with which we are familiar at the present time.

Personally I have great repugnance toward the fad. I believe in taking the broadest view possible of all methods and ideas by which we are able to render humanity a valuaable service. I stand here proud to say that I have rendered comfort and happiness to many a patient by the use of cement, amalgam, tin and gutta-percha.

Each year added to my professional life establishes more firmly the desire, and renders a stronger moral obligation, to perform the greatest service possible to my patients and by the use of methods and materials which shall give the patient the highest efficiency in service and comfort.

The many different phases of mechanical and manipulative ability existing with us individually makes it possible for us to reach similar results by many different methods, so in presenting this paper, if I am able to point out a single thing that may in any way add to the efficiency of anyone's accomplishments in this method of preserving the natural teeth, I shall feel highly repaid for my efforts.

Hand in hand with the advent of the porcelain art in dentistry came the gold inlay as we know it to-day. The gold inlay was first introduced many years ago as a sort of cast metal filling but it seems not to have been unsuccessfully handled, except by a very few men of the profession, and was not very generally used, with the progression of the day, the porcelain inlay has taken its present prominent place and with it as a sort of side development has again come the gold inlay, but with a better developed and cultivated knowledge for its construction and use which I feel will make it a permanent adjunct to our methods of saving teeth.

I scarcely believe that the men who occupy the ranks of the profession to-day are better men or are more conscientious in their work than were those of a generation ago, but I do believe the profession at the present time possess men, who, through better educational advantages and thorough study and a higher development of their mental faculties, are better able to think than our predecessors.

Through concentration of thought and orginal research along special lines, methods and ideas have been developed, superior ways of doing things and making sucess out of what has appeared to be failures to those of a few years ago.

Advancement in the profession is due very much to the character and high grade of young men who are selecting the profession of dentistry as their life work. Higher education and a collegiate training is better preparing a Doctor of Dental Surgery who is more capable of good, deep, rational thought, more capable of thinking scientifically and of prosecuting scientific research and investigation.

Gold and porcelain inlay work is but a mechanical advancement, which we have been making by such rapid strides till it looks as though we are nearly at the top. It would be wrong to say that this was due only to mechanical genius or manipulative skill, but we can truthfully say it is due to a combined union of genius, skill and scientific investigation.

We must not forget that our success dose not lie wholly within ourselves, there are those who have contributed materially to our welfare not by concentrating their thought along the line of how to preserve the tooth by means of the inlay, but of how to design the best and most practical instruments to assist in constructing the inlay, also to the manufacturer who is able to so admirably and beautifully produce our fine and delicate instruments.

Then again I must say sucess does not lie wholly within ourselves. In every phase of our professional life, we can not afford, not to come in contact with the other fellow, touch elbows as it were and make good use of some of his ideas. We progress also by the demand made upon us. The porcelain inlay has been brought about by the demand for the more esthetic, the gold inlay by a demand for a more resistant substance to withstand the ravages of decay and to better stand the strain of mastication.

Very important also for both is the demand made upon the up-to-date dentist of to-day by his patients who are now awake to the fact of more painless and less enervating methods. This argues strongly for the inlay because one of the great questions before us to-day is how to make our operation easiest for the patients.

It has been the great question with the public where to get dental service with the least amount of suffering. It will accomplish for the careful painstaking operator, who is striving for better fees as much as anyone thing I could mention. It is a demand that we are going to be compelled to meet.

The gold inlay I believe to be indicated in any cavity where we might use porcelain excepting in those cavities where we would place porcelain because of appearance.

It has been claimed by some that porcelain is the nearest to an ideal filling material for all classes of cavities. I must admit that for a majority of the cavities in the anterior teeth porcelain is nearly ideal, especially for those cavities which are very conspicuous, it certainly has no superior, but I must dispute its claim for superiority for large molar and many bicuspid restorations in masticating surfaces where the friability of porcelain gives us weak edges and thus necessitates its taking second place.

I have referred often to the porcelain inlay in this paper because the porcelain and the gold inlay are reciprocally related. The study of the methods of either results in the development of the other. We find in the practice of the one so many things so nearly identical that to be able to sucessfully use the one we must know much concerning the other.

The gold inlay is indicated in many of those places where we could only place cement, owing to the frail walls where the tooth has been so wasted away by the ravages of decay till there remains but little dentine underlying the thin wall of enamel. Here we are able to restore the strenght of the wall very materially by building up the body of the tooth with cement, then cutting back enamel margins to where they are heavy enough to resist decay, cutting a portion of our cavity in the cement, construct our inlay, set, finish and burnish the borders, and we have our tooth ably cared for. We have the strength of a cement filling and the durability of a gold filling. The gold inlay is suitable and much to be preferred in a great many of those cases where we have resorted to the crown.

It is by far superior to the crown where we are able to use it, for the reason that we are not in any way disturbing the surrounding tissues and they are left in their normal condition, and it is a fact that I have never been able to overthrow that I cannot improve upon nature around the neck of the tooth by the most skillful methods of crowning.

I say by most skillful methods because I wish to convey the idea of the inlay being superior to such methods, not alone to the ordinary ill fitting crown, with which we so often meet in daily practice'

As I have said before we regard the gold inlay as the best for very large cavities in the molars and bicuspids, the the occlusal surfaces, compound cavities in these teeth involving proximal surfaces or buccal surfaces and restoring cusps. The inlay is superior to the gold filling in its wear as it is so much harder. It will stand the wear of occlusion and mastication better, will not mar or scratch and when, in thin walls or edges through the changing of shape of the pure gold filling under the battering of occlusion causing a fracturing of enamel walls and margins, the inlay protects and renders the the tooth immune to such misfortune.

Do not understand me as advocating the gold inlay to the exclusion of the gold filling, each has its particular place where we may get our best desired results, but I might say, that I believe that some gold fillings would serve the tooth far better if we were to remove them in mass and reset in cement. I have a record of a similar case, a large cavity in a central had been refilled by a neighboring dentist the third time, the leverage was so great that it had been dislodged and patient wished me to refill. I experimented and reset the old filling in cement. That was eight years ago and the filling is still standing as an inlay, whereas its life as a filling had only been about one year.

The gold inlay may be practically utilized in some cases of bridge work to secure attachments where abutment teeth converge as we often find with the bicuspid and second or third molars, where patient has lost second bicuspid or first molar or both.

In the preparation of the cavity for the gold inlay we must avoid all undercuts that would in anyway distort the matrix in its removal from the cavity, the walls must diverge slightly from the parallel. This is not made absolutely necessary to get the closest fit as with the porcelain, as there is no matrix to be striped off from the finished inlay, but it aids removal of matrix from cavity.

Of course directly contrary to the principles of inserting the ordinary metalic fillings, the orifice of the cavity must be larger than the interior, a slight undercut is permissible at one side of the cavity if the other side is so beveled as to allow removal of matrix without distortion.

Inexperienced inlay workers often have had too much faith in the cement believing in its ability to hold the inlay to a saucer shaped cavity. Our scientific cement investigators and experimentors have not as yet given us that cement by which we can stick an inlay to almost a flat surface and make it stay there.

We find by experience that it is unnecessary to go to such extremes, we may easily shape our cavities with a view to just as much retention as it is possible to get. I never allow any chance to escape for getting more retentive form to secure all frictional holds possible by step cavities or otherwise which may assist in securing my inlay.

Be sure always to shape cavities by different walls and angles or by varied shapes of base so the matrix cannot move around, but will go to the exact place every time and cannot do otherwise.

All inlays should be so seated as to set on good flat bases and cavities so constructed that pressure will rather force the inlay into the cavity instead of out.

When it comes to the construction of the inlay we must consider that nearly every skillful man has his peculiar methods of doing things, and yet will arrive at the same results, and no doubt this will be one of the most important facts brought out in the discussion of this paper.

I have a preference in the procuring of the matrix, which

I obtained if it is possible by burnishing and swaging with unvulcanized rubber, gum camphor or sticky wax, or by use of the hollow pointed instruments, in which are inserted soft rubber points.

For matrix I use either the 1-1000 platinum, or pure gold No. 36 gauge.

A little trick I learned from the Jenkins people of folding a bit of silk chiffon over each side of the matrix before burnishing into the cavity has assisted me greatly in preventing, puncturing the matrix before getting to the bottom of the cavity.

Sometimes I take an impression of very large cavities, and like to use the Detroit Manufacturing Co's. modeling compound best. Fill the impression with cement, then by heating remove the compound; this gives me the cavity duplicated in cement into which I burnish and swage approximately, finishing in the tooth. If I desire to build up cusps I place a small amount of the compound in cavity and have patient close the teeth which gives the imprints of the opposing teeth, then remove and carve the cusps. This may also be done out of the mouth where the model containing the cement cavity has been mounted on an articulator with a bite of the opposite teeth. After having cusps and proper contours carved, I duplicate in gold plate by first filling the rubber ring, which accompanies Melotts outfit, with soft plaster, submerging the occluding and contour surfaces in the plaster and allowing it to set, then trimming away surplus plaster, removing compound, I have an impression of cusps and contour of my inlay. Now I duplicate this impression in fusible metal, by pressing a bit of the clay mixture ,or mouldine as it is called, into this plaster impression, turning it up-side-down and pressing the mouldine firmly upon the face of an anvil and then carefully removing the impression which comes away easily leaving the mouldine die sticking to the anvil, then I set one of the small rings, belonging to a Coats swager over it and pour with fusible metal, this gives me a counter die of cusps and contour surfaces of my inlay with outside measurements and by swaying in gold plate, 33 to 36 gauge, I have a cusps and contour which I adjust properly to matrix with sticky wax and open hole through bottom of matrix or through the contoured surface, and then invest and fill with solder. Always coat outside surfaces of matrix where you do not wish solder to flow with whiting or anti-flux.

If it is an inlay without cusps I fill matrix with either, or or both scraps 22k. gold or 22k. solder. Allow me to caution you, do not melt the entire mass of solder at once or you will be liable to get porosity also some claim you will get more shrinkage. I like to melt in piece by piece. When I desire a hollow inlay I place a piece of chalk or carbon, size desired, in bottom of matrix, pack fibre gold over and around it, flow solder over this, then when completed cut through bottom of matrix and remove this chalk or carbon leaving the hollow inlay.

I am very much like the physician who varies his medicine according to surroundings and circumstances. I hardly ever follow out the same rules or methods in every case, but vary them according to some peculiarity I desire in result.

Let me say as one important factor, be sure of a perfect matrix. The inlay should so fit the cavity so that the cement should form the merest film between inlay and tooth substance around the walls and margins. Over the base next to the pulp it is well to have more cement, as with the hollow inlay, to prevent thermal changes.

The value of the inlay as well as of a filling depends upon its weakest spot, a poor border, a poor anchorage or a misfit, just as the strength of a chain lies in its weakest link.

When you have carefully set your inlay be sure to burnish all edges carefully as this virtually seals the cement.

In summing up, let me say that we should be conservative in the use of the inlay as well as of any other treatment, be absolutely sure if possible of its giving success. The indiscriminate use of any method of material, no matter how much merit there is in it, will be very liable to injure you.

Wisdom and good judgement is always brought into use in deciding what method of restoration will give the best results. In any operation it is up to the operaton to decide when to use the inlay and when not to.

If the operation is a failure it will be because the operators judgement or skill, or both, are found wanting.

With the gold inlay we get all that is needed to stand the strain of mastication, we get a filling for the tooth which the fluids of the mouth or substances take into the mouth does not attack. It is chemically indestructable.

Its thermal non-conductivity gives much comfort to the patient, and by its close adaptability to the walls and margins it is almost a perfect seal to the cavity.

Because of the ease of insertion, without the use of the rubber dam, you will be informed by your patient that there is yet hope for a dentist beyond this life.

The gold inlay gives us durability, comfort to the patient, cleanliness, correct restoration of dental organs and saving of vital energy of both patient and operator.

DISCUSSION.

Dr. C. H. Worboys: On the gold inlay I am very much of the same opinion as Dr. Lyons. There are places to use and places not to use it. One of the points I have in mind where the gold inlay is used much better than a gold filling. Suppose we have a cavity, one of those mesio-occlusal cavities in an incisor, where you cut a groove across the incisal edge of the tooth to serve as a retainer. When you build your gold into that groove and down into a pit you make a wire as it were to hold your filling. I do not know of a poorer metal to make a wire of than pure gold for such a purpose. Now if you had a piece of gold wire and wanted to lengthen it a little you would put it on an anvil and tap it a little and and you find it stretches. Just so with the retaining wire on the incisal edge of the tooth, the masticating impacts of the occluding teeth will stretch your foil made wire and it does not push the small end of the filling along to make it deeper and tighter, but it pushes the body of the filling away from the tooth and gives a leaky filling, one of the best that you possibly ever made. The gold inlay for such cavities is better, for you have a harder metal because you use the fused and alloyed metal which is much harder and stronger.

You will have also a larger wire as you will cut the tooth away more to make the matrix and for that reason you will get a filling of greater strength. In the use of the gold inlay or any other inlay there is one thing that you will do that you would not do with the gold filling. You are not afraid to remove weak, irregular walls, because you know you can restore them easily. You can then use a stiff matrix and I think the consequences are that you get a better and stronger tooth, as there is nothing that is going to break down easily, and your filling is just as strong because there is a little more of it. You have restored a little more tooth, but it has taken very little more time.

The inlay can be used in almost any cavity of the mouth, but it is not always desirable to do so. No matter how badly decayed a tooth is, unless it is entirely gone and ready to crown, you can get some sort of retaining form, and with the inlay you need some sort of retaining form, if you can get it, besides the cement.

In the construction of the gold inlay I find some methods that I have used that are a little different than those the essayist has given you. In making a die for a gold inlay I take an impression of the cavity with a piece of gutta-percha. Around that I place a little piece of paper and pour in the low fusing metal. That gives a model of the tooth in the metal, place this model in the crown swedger on the sealing wax and burnish the gold or platinum approximately to it and then swedge. Any swedged matrix over any die or form is sure to fit, but I defy any man to burnish a piece of metal to fit anything. After the matrix has been swedged on the model remove the matrix and try it in the tooth and burnish it into place, or swedge it with cotton or rubber into the tooth. The next step that I use on large contour fillings for the restoration of a cusp with a hollow inlay for the grinding surface of the tooth, or for taking in any two sides of the tooth, is to take a bite in the mouth with modeling compound and then carve that right up to the matrix and put it in the swager, removing enough of the modeling compound to allow for the thickness of the gold.

Then placing this in the metallic impression, with the modeling compound in place, in the swedge and swedge the cusp plate right over it. Then fit the cap and matrix together, of course, removing the modeling compound, and solder the edges from the inside, protecting that part where you do not want the solder to flow.

There is one more point, and here I have had some failures. When you make a hollow gold inlay be sure and fill it with cement that is thick enough so that you can pack it in. Don't try to take a jiffy tube and fill the inlay with it, I have had two failures with it. They caved in. Don't try it.

DR. W. H. JACKSON: It is immaterial perhaps, nevertheless in reference to gold inlays, there are certain conditions to be considered before adapting them. There are conditions in which you cannot put in a metal filling or even porcelain inlay and preserve the life of the tooth. There are condition in which you cannot put in a gold filling without the tooth soon giving way. Many teeth are in such a condition that if you fill them in the course of a month or two the enamel will break away. This is a class of cases in which you will conserve the best interest of your patient, because an inlay will preserve the pulp. If you cut off a tooth and put on a jacket, or porcelain crown, you have approached the pulp nearer than you should have done in those cases; the gold set or inlay, is far superior to anything else that you can use in such cases. I have used them all the way from 1882 up to the present time. I have records on my books of them, and some of them I know have been in for 12 and 15 years. Some have gone into the graves; whether they would have lasted until now I do not know. If they are not properly made they are not as good as a gold crown. None of the cement should get to the surface so that the oral secretions can act upon it. You have to have pure gold in the matrix and drive it down perfectly true, and before your cement is set burnish your edge so that none of that cement is exposed. The result will be not be a success until you have done it that way.

If the secretions get to the cement it will dissolve out just as readily as from a porcelain set. I have 12 in one mouth, which I showed here last year, that are still intact and they are all as perfect as the day they were put in.

In grinding surfaces you have to use great care, because, if you leave too long a lapping edge in a short time that edge of gold is worn away leaving the cement exposed, and then you will get a dissolving of the cement. When the statement is made that porcelain has a tendency to prevent the cement from dissolving away and the tooth decaying, I would like to ask you to look at any of the Logan crowns you put in after five years, I do not care how many you look at. In your Logan crowns the cement is dissolved out in nine cases out of ten, a great portion of it inside of five years.

Why does not the porcelain there prevent the tooth from decaying just as they say it prevents the decay in the porcelain set.

A gold set, in order to be perfect, must come so near to the edge that it will stand almost perpendicular, then it will not wear away and expose the cement.

DR. LAND: In making inlays, I want to call your attention to the fact that I have some in use 18 years which is coincident with the invention of the porcelain process. I hold a patent on the gold inlay; this and the patent on porcelain inlays have both run out. I think that Dr. Robinson of Grand Rapids is the man, that after I had been refused a patent on the porcelain inlay, that secured it. I have practiced inlays ever since that time.

In having gold flow over a platinum matrix sucessfully I invariably paint the surface with precipitated gold, then I

can flow any karat of gold on it and bring it up to any form of contour desired. When pure or original gold is painted over the surface, it having a higher fusing point, the solder will flow to every point and cover it perfectly. All inlay methods originally called for a metal matrix that was swaged up with soft metal dies and every one of them can be made without puncturing, I do not care how deep they are.

DR. W. H. JACKSON: In the winter of 1885 there was a Dental Association Meeting at Ann Arbor, and in the clinic there was some operating done by different members. I operated, Dr. Field operated, Dr. E. C. Moore operated, and several others. Dr. Heroun, of Toledo, was filling a large molar tooth and I suggested to him that in such cases, where the walls were very thin I used a piece of thin gold and made it to fit the cavity and then filled it with solder and cemented it in the cavity. Dr. Land was present there at the time, and he says to me: What is that you say Doctor? I repeated my statement. After the Association adjourned that same evening Dr. Land came to my home and inquired about it further and we talked it over for an hour and a half. I could have for witnesses the persons that were there. On that same process three months afterwards Dr. Land had a patent, on the same thing I had attempted unsuccessfully to get one. I should like to ask Dr. Land if he did not come and talk to me on that subject.

*Answer.* Not until long after the process was invented.

This was in the winter of 1885 and you got yours the following May 20th. I had used the gold inlay at that time from 1881, and have my reords to show in my books. I told you I had done one at that time for the daughter of one of our dental dealers, and I also told Dr. Heroun how it was done. I admit that Dr. Land did put in the first fused porcelain set, but when he claimes the first gold inlay he is mistaken for it was somebody else.

DR. LYONS: The first thing that is due our patients is to give them the best service and most comfort it is possible to give, regardless of whether it is a gold filling or porcelain inlay. For the posterior teeth we are able to produce a piece of work by means of the gold inlay that absolutely fits the cavity and there is no stripping of the matrix and you do not have the thickness of the matrix to take into account.

If we extend out the gold matrix we are able to burinsh the edges and we can effectually seal the cavity. In regard to one point brought out by Dr. Worboys, that of getting the cusps, my experiences has caused me to differ from his method. In swaging the cusps by means of procuring the counter die in the fusible metal I do not have to take into account the thickness of the gold because I have the outside measurement. In making that die the thickness of the golds is taken into consideration and we get the counter die out side measurement instead of inside measurement.

